
JOURNAL INFORMATION
==============================
NLM Title Abbreviation: Sci China Life Sci
Journal ID: Sci China Life Sci
NLM Journal ID: 101529880

Science China. Life Sciences

ISSN: 1674-7305
EISSN: 1869-1889

ARTICLE INFORMATION
==============================
PMCID: PMC7088570
PMID: 25558864
DOI: 10.1007/s11427-014-4790-3
Article ID: 4790
Article version: 1
Subjects: Letter to the Editor

Do corticosteroids have a role in treating Ebola virus disease?

Xu Jun 1
Tan DingYu 1
Fu YangYang 1
Walline Joseph 2
Yu XueZhong yxzpumch@126.com
1
1 grid.410318.f 0000000406323409 Emergency Department, Peking Union Medical Hospital, Chinese Academy of Medical sciences, Beijing, 100730 China
2 grid.412359.8 0000000404573148 Division of Emergency Medicine, Department of Surgery, Saint Louis University Hospital, Saint Louis, Missouri 63110 USA
Electronic publication date: 2014 Dec 19
Print publication date: 2015
Volume: 58
Issue: 1
First page: 111
Last page: 113
Received 2014 Nov 3; Accepted 2014 Nov 23
Copyright: © The Author(s) 2014
License: This article is published under license to BioMed Central Ltd. Open Access This article is distributed under the terms of the Creative Commons Attribution License which permits any use, distribution, and reproduction in any medium, provided the original author(s) and source are credited.
License URL: https://creativecommons.org/licenses/by/4.0/
License: Open AccessThis article is distributed under the terms of the Creative Commons Attribution 2.0 International License (https://creativecommons.org/licenses/by/2.0), which permits unrestricted use, distribution, and reproduction in any medium, provided the original work is properly cited.
License URL: https://creativecommons.org/licenses/by/2.0/

Keywords: Hemorrhagic Fever, Dengue Hemorrhagic Fever, Severe Acute Respiratory Syndrome, Marburg Virus, Severe Acute Respiratory Syndrome Coronavirus
issue-copyright-statement: © Science in China Press and Springer-Verlag GmbH 2015

==============================

Dear Editor,

Humans have been fighting Ebola virus disease (EVD) since its first outbreak in 1976 in Yambuku village in the Democratic Republic of the Congo (previously Zaire). EVD is part of the Filoviridae family of viruses that includes Ebola and Marburg viruses. To date, EVD, one of the most deadly communicable diseases known to humans, has had 15 outbreaks in Africa. In 2014, the most severe and complicated outbreak yet swept through the West African countries of Guinea, Liberia, Nigeria, Senegal and Sierra Leone, with an initial case fatality rate of 70.8% [1]. As of 23 September 2014, with the situation seemingly still out of control, the number of suspected and confirmed cases was 6,553, with 3,083 deaths, including 208 healthcare workers.

The mechanisms by which EVD causes multisystem disease are poorly understood, and unfortunately, there are currently no licensed vaccines or drugs available for the treatment of EVD. Present treatment strategies for EVD are mainly supportive, while potentially effective vaccines and drugs (e.g., ZMapp) are still in varying stages of development. EVD is characterized by immune suppression and systemic inflammatory response syndrome (SIRS) that causes impairment of the vascular, coagulation, and immune systems, leading to shock and multiorgan failure. Some characteristics of SIRS pathophysiology resemble septic shock. In patients with septic shock, replacement doses of corticosteroids are thought to improve the mortality rate and reduce the duration of symptoms. Therefore, could a similar existing effect occur in those within EVD? Taking into account the role of significant inflammatory responses in EVD pathogenesis, we hypothesized that EVD patients might derive benefit from corticosteroid treatment to improve their clinical outcomes.

Monocytes, macrophages and immature dendritic cells, which are the initial sites for Ebola virus infection and replication, play a key role in the inflammatory processes during EVD. Infection of these cells leads to the uncontrolled release of pro-inflammatory cytokines and chemokines, including tumor necrosis factor-α (TNF-α), interferon (IFN), interleukin (IL)-2, IL-1β, IL-10 and macrophage inflammatory protein 1α (MIP-1α), as well as reactive oxygen and nitrogen radicals. These mediators attract increasing numbers of monocytes and macrophages as well as neutrophils, which release mediators of vasodilation and increase vascular permeability [2]. In particular, elevated levels of TNF-α followed by an increase in endothelial permeability might play an important role in the induction of circulatory shock during filovirus infection.

Failure of the early activation of T cells and the subsequent compromised adaptive immunity in EVD, including antibody and cytokine responses, is followed by the extensive apoptosis of blood leukocytes, and is closely associated with the fatal outcome of the disease. In the 2000 EVD outbreak in Uganda, a decrease in the number of circulating T lymphocytes was noted in people with fatal disease whereas cell counts were not reduced significantly in patients who survived the disease [3]. Impaired dendritic cell function

†Contributed equally to this work
*Corresponding author (email: yxzpumch@126.com)

caused by EVD may contribute to lymphocyte apoptosis. Furthermore, high levels of TNF-α produced by infected monocytes and macrophages also have an important role in lymphocyte apoptosis induced by TNF-related apoptosis-inducing ligand (TRAIL).

Elevated cytokine levels have been detected in the serum of patients with active EVD. Serum levels of pro-inflammatory cytokines in non-survivors were between five and 1000 times greater than that of convalescent or healthy individuals at the time of death [4]. Damage caused either directly by viral replication, or by the detrimental upregulation of pro-inflammatory cytokines, exacerbates the symptoms of disease and provides the platform for shock, multiorgan failure and death. Recovery from EVD requires an early, transient and controlled inflammatory response, which helps control virus replication and spread while simultaneously initiating the adaptive immune response.

An intact hypothalamic-pituitary-adrenal axis with effective intracellular glucocorticoid anti-inflammatory activity is essential for host survival following exposure to an infectious agent. Cortisol levels were reported to be elevated in patients in EVD. Viral infection can increase glucocorticoid concentrations during an acute infection to suppress pro-inflammatory responses that otherwise might be fatal. Cortisol levels ranged from 555 to 1110 nmol $L^{-1}$ in asymptomatic patients with Ebola infection compared with 133–377 nmol $L^{-1}$ in endemic control subjects [5].

However, adrenocortical infection and necrosis were also reported in humans and non-human primates infected with Ebola virus [6]. Inadequate intracellular glucocorticoid activity, referred to as critical illness-related corticosteroid insufficiency, typically results in an exaggerated pro-inflammatory response. Impaired steroid secretion leads to hypotension and sodium loss with hypovolemia, both of which have been reported in nearly all cases of EVD. Impairment of adrenocortical function by Ebola virus infection could therefore have an especially important role in the evolution of shock that typifies the late stages of EVD.

The anti-inflammatory actions of corticosteroids have been known since the 1940s. Because of their potent anti-inflammatory and diverse immuno-modulatory activities, corticosteroids have been widely used to treat many inflammatory and autoimmune diseases. Although there are controversies regarding their use, adjunctive corticosteroid treatment has preferential benefit in cases of severe infection where exaggerated inflammatory responses mediate the pathophysiology of disease (e.g., septic shock, severe pneumonia, avian influenza, and severe acute respiratory syndrome coronavirus). However, to our knowledge, no clinical studies have reported the efficacy of corticosteroids against EVD. Previous reports have focused on the treatment efficacy of corticosteroids against other types of viral hemorrhagic fever (VHF), such as Crimean-Congo hemorrhagic fever (CCHF), dengue hemorrhagic fever (DHF) and Hantaviruses hemorrhagic fever. In patients with CCHF and severe thrombocytopenia, high-dose methylprednisolone (10–30 mg $kg^{-1}$ per day) reduced fever, increased platelet and leukocyte counts, decreased internal bleeding and decreased dependency on blood products and reduced the mortality rate [7]. Despite the lack of clear survival benefit in patients with severe DHF, corticosteroids might help in reducing symptoms, improve well-being, shorten the time to platelet recovery or shorten the length of hospital stays [8].

Although different pathogens, such as EVD, CCHF, and DHF, have similar clinical features, such as fever, thrombocytopenia, hemorrhage, shock and multiple organ failure, treatment options for them are limited and are based on general supportive management. Similar to EVD, the pathogenesis of CCHF might be the result of direct injury of virus-infected tissues followed by the indirect effects of host immune responses, with abnormally high levels of cytokines and chemokines produced by activated monocytes and macrophages [9]. Early and well regulated inflammatory responses were associated with recovery from EVD. Although some DHF studies suggested that corticosteroids were not effective in reducing mortality and serious complications [10], it is important to consider the use of any potential effective treatment modalities because of the high mortality rate of EVD. Considering the past experiences with other VHFs, as well as inflammatory responses and impaired adrenocortical function in EVD, corticosteroids may be one of the better current options for treating patients with EVD.

Corticosteroids can be useful for treating infectious diseases when inflammation is particularly severe, but can be dangerous when the activation of inflammatory processes remains within the normal range. An important question is not just whether corticosteroid treatment will improve outcomes in various aspects of morbidity and mortality caused by EVD, but also whether it is safe. In addition to the increased risk of opportunistic infections and gastrointestinal bleeding caused by corticosteroids, enhancement of the current disease because of suppressed innate antiviral mechanisms is a primary concern. However, corticosteroids were already shown to be safe in some viral diseases, and there is no published evidence indicating the administration of corticosteroids causes the viral load to return to peak levels. In addition, no correlation between viral load levels and disease severity in patients with viral hemorrhagic fevers has been reported.

Because corticosteroids are not mentioned in the World Health Organization guidelines for the management of EVD, clinicians can only use them empirically, justifying their use based on the immunological basis of the life-threatening complications of the illness. The risk/benefit ratio of corticosteroids should be determined for each patient. Currently, there is no consistent recommendation regarding the type, dose and duration of corticosteroids for the treatment of EVD. Importantly the effectiveness of corticosteroid treatment may depend critically on the timing of drug administration. Immediate treatment following infection might help direct the developing immune response towards effective viral clearance while preventing severe symptoms in high-risk patients. Modest immunosuppression by corticosteroids might be safe for use in EVD patients as an adjunctive therapy in coordination with intensive supportive therapies.

*This work was supported by Ministry of Health Public Service Sector funded project (201302017).*

**Open Access** This article is distributed under the terms of the Creative Commons Attribution License which permits any use, distribution, and reproduction in any medium, provided the original author(s) and source are credited.

Contributed equally to this work

This article is published with open access at link.springer.com


References

1 WHO Ebola Response Team. Ebola virus disease in West Africa—the first 9 months of the epidemic and forward projections N Engl J Med 2014 10.1056/NEJMoa1411100 PMC4235004 25244186
2 Wong G Kobinger GP Qiu X Characterization of host immune responses in Ebola virus infections Expert Rev Clin Immunol 2014 10 781 790 10.1586/1744666X.2014.908705 24742338
3 Sanchez A Lukwiya M Bausch D Mahanty S Sanchez AJ Wagoner KD Rollin PE Analysis of human peripheral blood samples from fatal and nonfatal cases of Ebola (Sudan) hemorrhagic fever: cellular responses, virus load, and nitricoxide levels J Virol 2004 78 10370 10377 10.1128/JVI.78.19.10370-10377.2004 15367603 PMC516433
4 Wauquier N Becquart P Padilla C Human fatal Zaire Ebola virus infection is associated with an aberrant innate immunity and with massive lymphocyte apoptosis PLoS Negl Trop Dis 2010 4 10 10.1371/journal.pntd.0000837 PMC2950153 20957152
5 Leroy EM Baize S Debre P Lansoud-Soukate J Mavoungou E Early immune responses accompanying human asymptomatic Ebola infections Clin Exp Immunol 2001 124 453 460 10.1046/j.1365-2249.2001.01517.x 11472407 PMC1906073
6 Geisbert TW Hensley LE Larsen T Young HA Reed DS Geisbert JB Scott DP Kagan E Jahrling PB Davis KJ Pathogenesis of Ebola haemorrhagic fever in cynomolgus macaques: evidence that dendritic cells are early and sustained targets of infection Am J Pathol 2003 163 2347 2370 10.1016/S0002-9440(10)63591-2 14633608 PMC1892369
7 Sharifi-Mood B Alavi-Naini R Metanat M Mohammadi M Shakeri A Amjadi A Efficacy of high-dose methylprednisolone in patients with Crimean-Congo haemorrhagic fever and severe thrombocytopenia Trop Doct 2013 43 49 53 10.1177/0049475513486642 23796671
8 Rajapakse S Corticosteroids in the treatment of dengue illness Trans R Soc Trop Med Hyg 2009 103 122 126 10.1016/j.trstmh.2008.07.022 18789467
9 Erduran E Bahadir A Palanci N Gedik Y The treatment of Crimean-Congo hemorrhagic fever with high-dose methylprednisolone, intravenous immunoglobulin, and fresh frozen plasma J Pediatr Hematol Oncol 2013 35 e19 24 10.1097/MPH.0b013e3182706444 23018575
10 Shashidhara KC Murthy KAS Gowdappa HB Bhograj A Effect of high dose of steroid on platelet count in acute stage of dengue fever with thrombocytopenia J Clin Diagn Res 2013 7 1397 1400 23998074 10.7860/JCDR/2013/6135.3143 PMC3749644
